# Mortality Outcomes in a Large Population with and without Covert Cerebrovascular Disease

**DOI:** 10.14336/AD.2024.0211

**Published:** 2024-02-11

**Authors:** Úna Clancy, Eric J. Puttock, Wansu Chen, William Whiteley, Ellen M. Vickery, Lester Y. Leung, Patrick H. Luetmer, David F. Kallmes, Sunyang Fu, Chengyi Zheng, Hongfang Liu, David M. Kent

**Affiliations:** ^1^Centre for Clinical Brain Sciences, Edinburgh Imaging, and UK Dementia Research Institute, University of Edinburgh, Edinburgh EH16 4SB, United Kingdom.; ^2^Department of Research and Evaluation, Kaiser Permanente Southern California, Pasadena, California, USA.; ^3^Predictive Analytics and Comparative Effectiveness Center, Tufts Medical Center, Boston, Massachusetts, USA.; ^4^Department of Neurology, Tufts Medical Center, Boston, Massachusetts, USA.; ^5^Department of Radiology, Mayo Clinic, Rochester, Minnesota, USA.; ^6^Center for Translational AI Excellence and Applications in Medicine, University of Texas Health Science Center, Houston, Texas, USA

**Keywords:** mortality, cerebral infarction, small vessel disease, magnetic resonance imaging, aging

## Abstract

Covert cerebrovascular disease (CCD) is frequently reported on neuroimaging and associates with increased dementia and stroke risk. We aimed to determine how incidentally-discovered CCD during clinical neuroimaging in a large population associates with mortality. We screened CT and MRI reports of adults aged ≥50 in the Kaiser Permanente Southern California health system who underwent neuroimaging for a non-stroke clinical indication from 2009-2019. Natural language processing identified incidental covert brain infarcts (CBI) and/or white matter hyperintensities (WMH), grading WMH as mild/moderate/severe. Models adjusted for age, sex, ethnicity, multimorbidity, vascular risks, depression, exercise, and imaging modality. Of n=241,028, the mean age was 64.9 (SD=10.4); mean follow-up 4.46 years; 178,554 (74.1%) had CT; 62,474 (25.9%) had MRI; 11,328 (4.7%) had CBI; and 69,927 (29.0%) had WMH. The mortality rate per 1,000 person-years with CBI was 59.0 (95%CI 57.0-61.1); with WMH=46.5 (45.7-47.2); with neither=17.4 (17.1-17.7). In adjusted models, mortality risk associated with CBI was modified by age, e.g. HR 1.34 [1.21-1.48] at age 56.1 years vs HR 1.22 [1.17-1.28] at age 72 years. Mortality associated with WMH was modified by both age and imaging modality e.g., WMH on MRI at age 56.1 HR = 1.26 [1.18-1.35]; WMH on MRI at age 72 HR 1.15 [1.09-1.21]; WMH on CT at age 56.1 HR 1.41 [1.33-1.50]; WMH on CT at age 72 HR 1.28 [1.24-1.32], vs. patients without CBI or without WMH, respectively. Increasing WMH severity associated with higher mortality, e.g. mild WMH on MRI had adjusted HR=1.13 [1.06-1.20] while severe WMH on CT had HR=1.45 [1.33-1.59]. Incidentally-detected CBI and WMH on population-based clinical neuroimaging can predict higher mortality rates. We need treatments and healthcare planning for individuals with CCD.

## INTRODUCTION

Covert cerebrovascular disease (CCD) is commonly discovered incidentally on routine clinical neuroimaging and is associated with a substantial increase in the risk of future stroke and dementia [1, 2]. Covert cerebrovascular disease (CCD) refers to incidentally-detected small or large vessel cerebrovascular disease that is not associated with a history of stroke or cognitive impairment, e.g. dementia. However, CCD is far from benign and carries an increased risk of dementia and stroke [2-4]. Since CCD is highly prevalent [5, 6], especially in advancing age, identifying the full implications of CCD outcomes is necessary. This facilitates health service planning, clinically meaningful prognostication, and trial design by identifying timelines for relevant endpoints.

Associations between mortality and silent cerebrovascular disease has been established in research populations using MRI [3], e.g. severe white matter hyperintensities (WMH) are associated with two-fold brain infarcts with 1.64-fold mortality risk. However, apart from previous work focusing on smaller stroke [7-12], memory clinic [13] and community-dwelling [14-22] populations, it is less clear how incidentally detected CCD associates with mortality outcomes in a large, real-world, population-based sample who have undergone neuroimaging for clinical indications, using CT or MRI.

This study aims to examine how incidentally detected CCD in a real-world setting, i.e. during routine neuroimaging, prognosticates future mortality risk.

## MATERIALS AND METHODS

Population and setting details have been published previously [2, 4]. The present analysis includes patients aged ≥50 years who were Kaiser Permanente Southern California (KPSC) health system enrolees undergoing neuroimaging for any non-stroke, non-cognitive clinical indication between 2009-2019. Cognitive indications included dementia, confusion, disorientation, altered mental status. Clinical neuroimaging indications are described elsewhere [2]. The main indications included headache, dizziness, auditory, vascular risk factors, and falls. We used the index scan, defined as the first available scan since KPSC enrolment. We defined follow-up as the interval between 60 days post-neuroimaging and the earliest of: (a) mortality (b) disenrollment from KPSC, or (c) study end date.

We excluded patients with a history of stroke, TIA, dementia, neurovascular procedures, or other neurological conditions at index scan date. We excluded patients whose follow-up was <60 days after the index scan including those receiving a new stroke or dementia diagnosis during this period (Supplementary Fig. 1).

We defined CCD as incidentally-discovered covert brain infarcts (CBI) and/or WMH. We used a Natural Language Processing (NLP) algorithm devised by Mayo Clinic and Tufts Medical Center to screen CT and MRI reports using an open-source pipeline in MedTagger and using both generic and task-specific NLP which incorporated words and phrases relating to WMH and CBI, CBI subtype (subcortical vs cortical) and WMH severity (none, mild, moderate, severe, or unspecified). We validated the NLP algorithm by comparing NLP results against the gold standard generated by the two trained resident neuroradiologists with F scores of 0.91 for WMH and 0.90 for CBI [2]. Indeed, agreement between the NLP and the trained readers was similar to agreement between the trained readers [23] and agreement between the NLP and direct neuroradiological review was similar to agreement between two neuroradiologists directly reviewing the scans [24].

### Statistical analysis

We used Kaplan-Meier plots to display mortality-free survival in patients with vs without CCD, defined as presence vs absence of CBI or WMH. We assessed survival differences between patients with vs without CCD using the log-rank test. We calculated overall and modality- and WHM severity-stratified crude incidence rates, with corresponding 95% confidence interval reported as per 1,000-person years of follow-up. We examined crude and adjusted associations of WMH severity with mortality using Cox proportional hazards regression models, adjusting for age, sex, ethnicity, Charlson comorbidity index, vascular risk factors including smoking, mean systolic blood pressure over one-year, antiplatelet use, statin use, depression, hours/week exercise, BMI, imaging modality (CT/MRI) and age*WMH, age*CBI, and modality-WMH. We chose these variables a priori as key predictors of global mortality. (Evaluation IfHMa. Global Burden of Disease Collaborative Network: Global Burden of Disease Study 2019 Results 2020) Multiple imputation with 10 imputed databases was used to account for missingness in the continuous variables.

Analyses were performed using SAS Version 9.4 and R Version 3.6.0.

The study protocol was approved by Tufts Medical Center and KPSC Institutional Review Boards which waived the need for informed consent as data were anonymized.

## RESULTS

**Figure 1.**
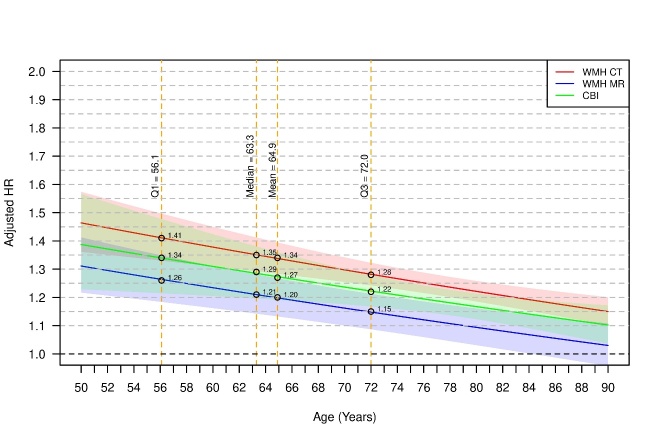
Mortality associated with CBI and WMH according to both age and imaging modality.

The present analysis includes 241,028 KPSC cohort members (Supplemetary Fig. 1). The mean age was 64.9 years (SD=10.4) and mean follow-up was 4.46 years (Table 1). 178,554 (74.1%) had CT and 62,474 (25.9%) had MRI. 11,328 (4.7%) had CBI; 69,927 (29.0%) had WMH; and 166,057 (68.8%) had neither.

Both CBI and WMH were associated with a substantial increase in mortality compared to patients without these findings (Supplementary Fig. 2). The mortality rate per 1,000 person-years was 59.0 (95% CI 57.0-61.1) in patients with CBI; 46.5 (45.7-47.2) in patients with WMH and 17.4 (17.1-17.7) in patients with neither (Table 2). The unadjusted mortality Hazard Ratio (HR) was 2.42 (95% CI 2.33-2.51) for CBI and 2.56 (2.50-2.62) for WMH (Supplementary Table 1).

In adjusted models, the mortality risk associated with CBI on CT/MRI (Fig. 1) was modified by age (e.g. HR 1.34 [1.21-1.48] at age 56.1 years vs HR 1.22 [1.17-1.28] at age 72 years). Mortality associated with WMH was modified by both age and imaging modality (Fig. 1, e.g. WMH on MRI at age 56.1 HR 1.26 [1.18-1.35]; WMH on MRI at age 72 HR 1.15 [1.09-1.21]; WMH on CT at age 56.1 HR 1.41 [1.33-1.50]; WMH on CT at age 72 HR 1.28 [1.24-1.32], vs. patients without CBI or without WMH, respectively]); Supplementary Table 1 and Supplementary Table 2.

**Table 1:** Patient demographic and clinical characteristics at baseline.

Patient characteristics	Entire cohort	Subset: patients with id-CBI	Subset: patients with id-WMH	Subset: patients without id-CCD
**N**	241,028	11,328	69,927	166,057
**Demographics**				
**Age, mean (SD)**	64.9 (10.42)	72.2 (10.79)	70.7 (10.77)	62.3 (9.17)
**Female**	147,797 (61.3%)	6,447 (56.9%)	42,656 (61.0%)	102,249 (61.6%)
**Race/ ethnicity**				
**Asian and Pacific Islander**	28,353 (11.8%)	1,256 (11.1%)	8,030 (11.5%)	19,755 (11.9%)
**African American**	27,301 (11.3%)	1,643 (14.5%)	7,821 (11.2%)	18,741 (11.3%)
**Hispanic**	78,134 (32.4%)	2,869 (25.3%)	17,417 (24.9%)	59,243 (35.7%)
**Multiple/Other/Unknown**	3,999 (1.7%)	134 (1.2%)	990 (1.4%)	2,951 (1.8%)
**Non-Hispanic white**	103,241 (42.8%)	5,426 (47.9%)	35,669 (51.0%)	65,367 (39.4%)
**Stroke risk factors**				
**Atrial fibrillation**	14,073 (5.8%)	1,314 (11.6%)	6,372 (9.1%)	7,247 (4.4%)
**Carotid atherosclerosis**	2,312 (1.0%)	206 (1.8%)	1,125 (1.6%)	1,120 (0.7%)
**Congestive heart failure**	11,448 (4.8%)	1,214 (10.7%)	5,228 (7.5%)	5,782 (3.5%)
**Coronary artery disease**	25,790 (10.7%)	2,162 (19.1%)	10,324 (14.8%)	14,664 (8.8%)
**Diabetes mellitus**	62,531 (25.9%)	3,995 (35.3%)	20,383 (29.2%)	40,475 (24.4%)
**Hypercholesterolemia**	163,795 (68.0%)	8,505 (75.1%)	51,024 (73.0%)	109,079 (65.7%)
**Hypertension**	145,526 (60.4%)	8,941 (78.9%)	49,758 (71.2%)	92,052 (55.4%)
**Peripheral arterial disease**	10,538 (4.4%)	1,067 (9.4%)	4,749 (6.8%)	5,433 (3.3%)
**Tobacco use (ever)**				
**Yes**	111,263 (46.2%)	5,998 (53.0%)	35,122 (50.2%)	73,545 (44.3%)
**No**	128,992 (53.5%)	5,294 (46.7%)	34,670 (49.6%)	91,899 (55.3%)
**Unknown**	773 (0.3%)	36 (0.3%)	135 (0.2%)	613 (0.4%)
**Number of stroke risk factors**	2.3 (1.47)	2.9 (1.59)	2.6 (1.52)	2.1 (1.42)
**Systolic blood pressure, mean (SD)^1^**	129.3 (13.17)	132.7 (13.85)	131.4 (13.09)	128.4 (13.07)
**Modality**				
**CT**	178,554 (74.1%)	9,041 (79.8%)	36,901 (52.8%)	137,106 (82.6%)
**MRI**	62,474 (25.9%)	2,287 (20.2%)	33,026 (47.2%)	28,951 (17.4%)
**Antiplatelet use**	10,985 (4.6%)	753 (6.7%)	3,927 (5.6%)	6,763 (4.1%)
**Statin use**	103,567 (43.0%)	6,022 (53.2%)	34,996 (50.1%)	66,020 (39.8%)
**Depression**	47,053 (19.5%)	2,189 (19.3%)	13,713 (19.6%)	32,318 (19.5%)
**Exercise (hour/per week), mean (SD)^1^**	1.7 (2.53)	1.5 (2.39)	1.7 (2.50)	1.8 (2.54)
**BMI (kg/m^2^), mean (SD)^1^**	28.7 (6.04)	27.9 (5.94)	27.9 (5.83)	29.0 (6.09)
**Charlson comorbidity index**				
**0**	114,424 (47.5%)	3,732 (32.9%)	26,109 (37.3%)	86,336 (52.0%)
**1**	49,170 (20.4%)	2,065 (18.2%)	13,651 (19.5%)	34,550 (20.8%)
**2**	31,590 (13.1%)	1,780 (15.7%)	10,635 (15.2%)	20,209 (12.2%)
**3-4**	26,261 (10.9%)	2,046 (18.1%)	10,795 (15.4%)	14,720 (8.9%)
**5+**	19,583 (8.1%)	1,705 (15.1%)	8,737 (12.5%)	10,242 (6.2%)
**id-CBI**	11,328 (4.7%)	NA	6,284 (9.0%)	NA
**id-WMH**	69,927 (29.0%)	6,284 (55.5%)	NA	NA
**id-CCD**	74,971 (31.1%)	NA	NA	NA

id-CBI: incidentally-discovered covert brain infarction; id-WMH: incidentally-discovered white matter hyperintensities; id-CCD: incidentally-discovered covert cerebrovascular disease comprising id-CBI and id-WMH; MRI: Magnetic resonance Imaging; BMI: Body Mass Index; SD: standard deviation. ^1^mean SBP was missing in 8,843 patients (3.7% of the cohort); exercise in 34,118 patients (14.2%); BMI in 2,303 patients (1%).

**Figure 2.**
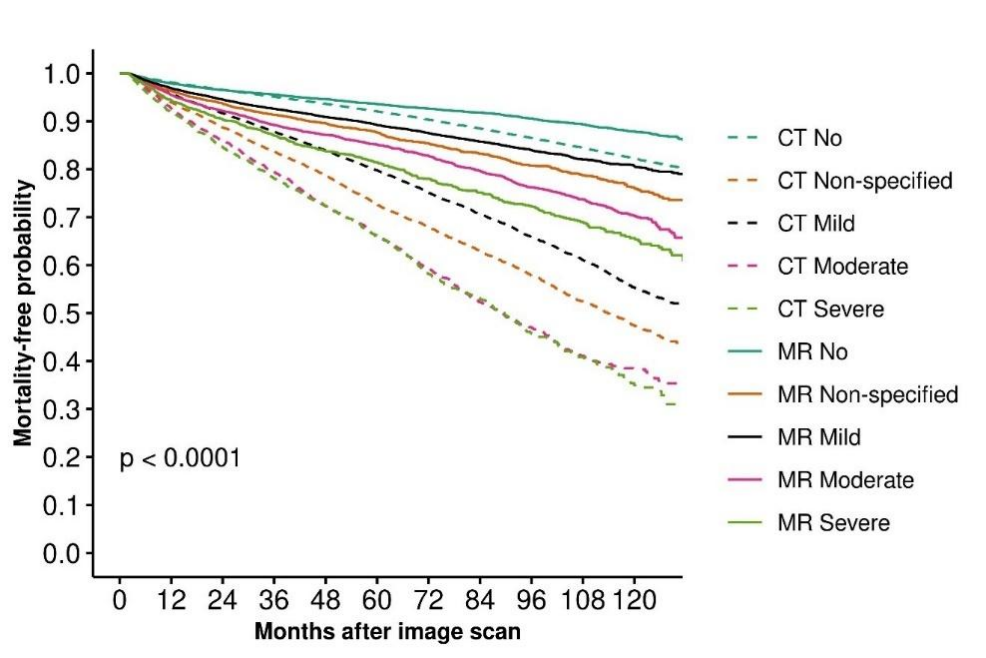
**Kaplan-Meier plot of Mortality-free probability according to id-WMH severity and imaging modality**. WMH = white matter hyperintensities; id = incidentally-discovered; CT = computed tomography; MRI = magnetic resonance imaging; No = no; id-WMH; Non-specified = scan report did not specify presence or absence of id-WMH; Mild = scan reported mild id-WMH; Moderate = scan reported moderate id-WMH; Severe = scan reported severe id-WMH.

Compared to the referent category of no WMH on MRI, increasing WMH severity was associated with higher mortality within each category of imaging modality (MRI or CT) (Fig. 2; Supplementary Table 3). Moreover, CCD-mortality and WMH severity associations were stronger in patients who underwent CT vs. MRI. For example, with severe WMH on CT, HR=1.45 [1.33-1.59] vs. mild WMH on CT, HR=1.18 [1.12-1.25]; severe WMH on MRI, HR=1.24 [1.11-1.38] vs mild WMH on MRI, HR=1.13 [1.06-1.20] (Supplementary Table 3; Fig. 2). To translate this, an individual with severe WMH on MRI has a 24% increased mortality rate and severe WMH on CT a 45% increased mortality rate, compared with individuals with no WMH on MRI.

**Figure 3.**
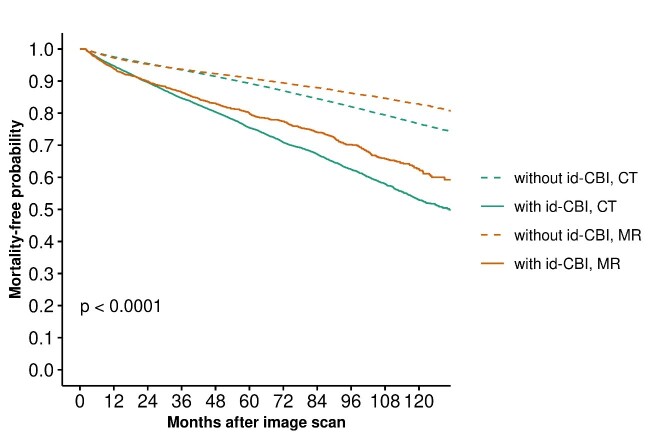
**This plot demonstrates the decreasing influence of covert cerebrovascular disease findings with increasing age based on a Cox proportional hazards model**. id-CBI by modality. id = incidentally-discovered; CBI = covert brain infarcts; MR = Magnetic Resonance Imaging; CT = computed tomography imaging

Figure 3 and Supplementary Figure 3 show mortality related to incidentally-detected CBI by (a) imaging modality and (b) CBI location (subcortical vs cortical) by modality. Supplementary Table 4 shows crude and adjusted effects on mortality for all risk factors. Supplementary Table 5 shows mortality rate with follow-up >1 year. Supplementary Tables 6 and 7 show cause-specific and crude and adjusted effects on mortality rate.

**Table 2:** Mortality rate overall and in subgroups (n=241,028).

Patient Characteristics	Patients with id-CBI (n=11,328)			Patient with id-WMH (n=69,927)			Patients with both id-CBI and id-WMH (n=6,284)			Patients without id-CCD^a^ (n=166,057)		
	Avg f/u, yrs	No. events	Mortality rate (95% CI)	Avg f/u, yrs	No. events	Mortality rate (95% CI)	Avg f/u, yrs	No. events	Mortality rate (95% CI)	Avg f/u, yrs	No. events	Mortality rate (95% CI)
**All**	4.57	3,057	59.0 (57.0,61.1)	4.30	13,981	46.5 (45.7,47.2)	4.27	2,033	75.7 (72.5,79.0)	4.53	13,108	17.4 (17.1,17.7)
**Age group (yrs)**												
**50-59**	4.65	129	16.1 (13.5,19.1)	4.41	712	12.3 (11.4,13.2)	4.45	60	25.6 (19.7,32.8)	4.43	2,595	7.4 (7.1,7.7)
**60-69**	4.78	443	31.0 (28.2,34.0)	4.41	2,243	25.2 (24.2,26.3)	4.42	236	38.9 (34.2,44.1)	4.59	3,687	15.1 (14.6,15.6)
**70-79**	4.87	1,030	57.6 (54.1,61.2)	4.45	4,583	47.6 (46.2,49.0)	4.57	655	64.1 (59.3,69.1)	4.76	3,925	32.5 (31.5,33.5)
**80+**	3.94	1,455	125.3 (119.0,131.9)	3.85	6,443	111.6 (108.9,114.4)	3.82	1,082	131.5 (123.9,139.5)	4.31	2,901	83.0 (80.0,86.1)
**Sex**												
**Female**	4.83	1,581	50.8 (48.3,53.3)	4.44	7,834	41.4 (40.5,42.3)	4.52	1,074	66.9 (63.0,71.0)	4.64	6,922	14.6 (14.3,14.9)
**Male**	4.23	1,476	71.4 (67.8,75.1)	4.09	6,147	55.0 (53.7,56.4)	3.96	959	88.8 (83.3,94.6)	4.35	6,186	22.3 (21.7,22.8)
**Race/ ethnicity**												
**Non-Hisp. white**	4.50	1,677	68.8 (65.5,72.1)	4.33	8,264	53.5 (52.4,54.7)	4.22	1,167	85.8 (80.9,90.8)	4.62	6,791	22.5 (22.0,23.1)
**Asian/PI**	4.81	252	41.7 (36.8,47.1)	4.44	1,144	32.1 (30.3,34.0)	4.36	153	51.0 (43.4,59.5)	4.75	1,119	11.9 (11.2,12.6)
**African Am.**	4.80	482	61.1 (55.8,66.7)	4.42	1,822	52.7 (50.4,55.2)	4.46	323	80.0 (71.7,89.1)	4.81	1,759	19.5 (18.6,20.4)
**Hispanic**	4.53	629	48.3 (44.7,52.2)	4.19	2,654	36.4 (35.0,37.8)	4.30	381	63.6 (57.4,70.2)	4.32	3,349	13.1 (12.7,13.5)
**Other^b^**	3.43	17	37.0 (22.3,57.8)	3.43	97	28.5 (23.3,34.7)	2.86	9	41.5 (19.0,78.7)	3.58	90	8.5 (6.9,10.4)
**Imaging Modality**												
**CT**	4.57	2,542	61.5 (59.1,63.9)	4.15	9,766	63.8 (62.6,65.1)	4.21	1,606	84.9 (80.9,89.2)	4.53	11,286	18.2 (17.8,18.5)
**MRI**	4.58	515	49.2 (45.1,53.6)	4.48	4,215	28.5 (27.6,29.4)	4.44	427	53.7 (48.8,59.0)	4.50	1,822	14.0 (13.4,14.6)

PI: Pacific islander; id-CBI: incidentally-discovered covert brain infarction; id-WMH: incidentally-discovered white matter hyperintensities; id-CCD: incidentally-discovered covert cerebrovascular disease. ^a^id-CBI or id-WMH; ^b^Multiple, other or unknown race/ethnicity.

## DISCUSSION

Incidentally-discovered CBI and WMH on routine clinical neuroimaging herald higher mortality risk compared to patients without these features, after adjusting for confounders. Moreover, mortality rate increases according to WMH severity, regardless of imaging modality used.

Our findings add to the emerging evidence that these incidental findings have important clinical consequences [2, 4, 25, 26], highlighting this population as a target for intervention. While incidentally-discovered CCD is a strong prognostic biomarker for stroke, dementia and now mortality, the extent of direct causal contributions should be addressed in studies with long term follow-up and/or randomized studies of interventions targeting vascular risk. Most incidental CCD, including CBI, most of which are subcortical [27], and WMH [28], results from small vessel disease. While progression of CCD may lead to stroke, dementia and increasing frailty, which all might contribute to higher mortality, it may coexist with systemic vascular disease and other comorbidities.

Our finding of higher mortality in patients with CCD, particularly WMH, who underwent CT vs MRI recapitulates our prior findings for stroke and dementia [2, 4]. In previous work, patients with “mild” WMH on CT had similar or even slightly worse outcomes than those found to have “severe” WMH on MRI. This may be because CT scans are less sensitive than MRI for detecting disease. Alternatively, patients undergoing CT may be frailer.

Our study has limitations inherent to our use of real-world data. Incomplete or inaccurate data may add noise. Some binary data elements, coded as “absent”, may instead reflect missingness. WMH severity was missing from neuroimaging reports in 8% of MRI and 6% of CT, reflecting report quality in routine care. Causal inference cannot be drawn from observational studies like this without strong unverifiable assumptions and our study involves patients with clinically-indicated scans, rather than population-based cohorts, which might be influenced by “collider” (i.e., selection) bias [29]. Nevertheless, real-world data is our study’s main strength, reflecting true prognostic value in large populations. We are not aware of any published population-based studies of mortality and CCD at this level of scale, i.e. almost a quarter of a million people. This population-level data allows real-world clinical translation to populations attending clinical neuroimaging for diverse indications, and the findings are particularly applicable to future large-scale public health initiatives in this population where reduction in population mortality rates should be a goal.

The benefits of large real-world datasets are inevitably accompanied by limitations. Although mortality is accurately coded, inaccurate coding of health conditions assessed as covariates in the present analysis is a possibility. Moreover, coding of CCD imaging abnormalities is dependent on the content of the radiology reports on which the NLP is based, so it is possible that we may have underestimated the prevalence of CCD in this population. However, our NLP algorithm has been tested against the gold standard of visual assessment by two radiologists [23, 24].

Our definition of covert cerebrovascular disease as cerebrovascular disease without a history of stroke or cognitive impairment accommodates patients with subtle neurocognitive symptoms such as gait decline resulting in falls. This is in keeping with the ESO Guideline on covert cerebral small vessel disease [30], which represents a pragmatic diagnostic category for both research and clinical purposes since in practice it is difficult to determine the boundaries of these symptoms and their causes, which are often multifactorial and not always clearly attributable to a cerebrovascular origin. Taken together, our findings should be interpreted carefully in light of our CCD definition and the populations that this definition applies to.

Our findings have important clinical relevance. Incidentally-discovered covert brain infarcts and white matter hyperintensities on routinely-obtained clinical neuroimaging herald higher mortality risk compared to patients without these features. More severe white matter hyperintensities associates with higher mortality than mild or no white matter hyperintensities, regardless of imaging modality. At minimum, it is essential to inform patients of these findings, which is frequently not done, and to encourage lifestyle choices targeted to improving cerebrovascular health as outlined in small vessel disease guidelines [30], such as regular exercise, smoking cessation, healthy diet including reduced salt intake, and also to evaluate whether any additional medical interventions might be necessary, such as improved blood pressure or lipid level management. Finally, we anticipate that as more attention is paid to this entity, there will be more research and the evidence base for the management of CCD will improve. Moreover, our findings assist individual clinicians in counselling their patients about prognosis and risk, add impetus for large-scale public health interventions in this routinely imaged population, and also add weight to research funders to prioritise trials that prevent small and large vessel disease progression.

Incidentally-detected CBI and WMH on clinical neuroimaging in a real-world population heralds higher mortality rates. Mortality risk rises with increasing WMH severity regardless of imaging modality. This routine health data could help guide prognostication and health service planning.

## Supplementary Materials

The Supplementary data can be found online at: www.aginganddisease.org/EN/10.14336/AD.2024.0211.



## References

[1] WardlawJM, SmithC, DichgansM (2019). Small vessel disease: mechanisms and clinical implications. Lancet Neurol, 18:684-696.31097385 10.1016/S1474-4422(19)30079-1

[2] KentDM, LeungLY, ZhouY, LuetmerPH, KallmesDF, NelsonJ, et al. (2023). Association of Incidentally Discovered Covert Cerebrovascular Disease Identified Using Natural Language Processing and Future Dementia. J Am Heart Assoc, 12:e027672.36565208 10.1161/JAHA.122.027672PMC9973577

[3] DebetteS, SchillingS, DuperronMG, LarssonSC, MarkusHS (2019). Clinical Significance of Magnetic Resonance Imaging Markers of Vascular Brain Injury: A Systematic Review and Meta-analysis. JAMA Neurol, 76:81-94.30422209 10.1001/jamaneurol.2018.3122PMC6439887

[4] KentDM, LeungLY, ZhouY, LuetmerPH, KallmesDF, NelsonJ, et al. (2021). Association of Silent Cerebrovascular Disease Identified Using Natural Language Processing and Future Ischemic Stroke. Neurology, 97:e1313-e1321.34376505 10.1212/WNL.0000000000012602PMC8480402

[5] VermeerSE, KoudstaalPJ, OudkerkM, HofmanA, BretelerMM (2002). Prevalence and risk factors of silent brain infarcts in the population-based Rotterdam Scan Study. Stroke, 33:21-25.11779883 10.1161/hs0102.101629

[6] de LeeuwFE, de GrootJC, AchtenE, OudkerkM, RamosLM, HeijboerR, et al. (2001). Prevalence of cerebral white matter lesions in elderly people: a population based magnetic resonance imaging study. The Rotterdam Scan Study. J Neurol Neurosurg Psychiatry, 70:9-14.11118240 10.1136/jnnp.70.1.9PMC1763449

[7] AndersenSD, LarsenTB, Gorst-RasmussenA, YavarianY, LipGY, BachFW (2017). White Matter Hyperintensities Improve Ischemic Stroke Recurrence Prediction. Cerebrovasc Dis, 43:17-24.27750251 10.1159/000450962

[8] AppelrosP, SamuelssonM, LindellD (2005). Lacunar Infarcts: Functional and Cognitive Outcomes at Five Years in Relation to MRI Findings. Cerebrovascular Diseases, 20:34-40.15942172 10.1159/000086202

[9] FuJH, LuCZ, HongZ, DongQ, LuoY, WongKS (2005). Extent of white matter lesions is related to acute subcortical infarcts and predicts further stroke risk in patients with first ever ischaemic stroke. J Neurol Neurosurg Psychiatry, 76:793-796.15897500 10.1136/jnnp.2003.032771PMC1739660

[10] PutaalaJ, HaapaniemiE, KurkinenM, SalonenO, KasteM, TatlisumakT (2011). Silent brain infarcts, leukoaraiosis, and long-term prognosis in young ischemic stroke patients. Neurology, 76:1742-1749.21576692 10.1212/WNL.0b013e31821a44ad

[11] OksalaNK, OksalaA, PohjasvaaraT, VatajaR, KasteM, KarhunenPJ, ErkinjunttiT (2009). Age related white matter changes predict stroke death in long term follow-up. J Neurol Neurosurg Psychiatry, 80:762-766.19237385 10.1136/jnnp.2008.154104

[12] YamauchiH, FukudaH, OyanagiC (2002). Significance of white matter high intensity lesions as a predictor of stroke from arteriolosclerosis. J Neurol Neurosurg Psychiatry, 72:576-582.11971040 10.1136/jnnp.72.5.576PMC1737875

[13] HennemanWJ, SluimerJD, CordonnierC, BaakMM, ScheltensP, BarkhofF, van der FlierWM (2009). MRI biomarkers of vascular damage and atrophy predicting mortality in a memory clinic population. Stroke, 40:492-498.19109551 10.1161/STROKEAHA.108.516286

[14] BokuraH, KobayashiS, YamaguchiS, IijimaK, NagaiA, ToyodaG, et al. (2006). Silent brain infarction and subcortical white matter lesions increase the risk of stroke and mortality: a prospective cohort study. J Stroke Cerebrovasc Dis, 15:57-63.17904049 10.1016/j.jstrokecerebrovasdis.2005.11.001

[15] ConijnMM, KloppenborgRP, AlgraA, MaliWP, KappelleLJ, VinckenKL, et al. (2011). Cerebral small vessel disease and risk of death, ischemic stroke, and cardiac complications in patients with atherosclerotic disease: the Second Manifestations of ARTerial disease-Magnetic Resonance (SMART-MR) study. Stroke, 42:3105-3109.21868739 10.1161/STROKEAHA.110.594853

[16] WindhamBG, DeereB, GriswoldME, WangW, BezerraDC, ShibataD, et al. (2015). Small Brain Lesions and Incident Stroke and Mortality: A Cohort Study. Ann Intern Med, 163:22-31.26148278 10.7326/M14-2057PMC4551397

[17] DebetteS, BeiserA, DeCarliC, AuR, HimaliJJ, Kelly-HayesM, et al. (2010). Association of MRI markers of vascular brain injury with incident stroke, mild cognitive impairment, dementia, and mortality: the Framingham Offspring Study. Stroke, 41:600-606.20167919 10.1161/STROKEAHA.109.570044PMC2847685

[18] IkramMA, VernooijMW, VroomanHA, HofmanA, BretelerMM (2009). Brain tissue volumes and small vessel disease in relation to the risk of mortality. Neurobiol Aging, 30:450-456.17766013 10.1016/j.neurobiolaging.2007.07.009

[19] KullerLH, ArnoldAM, LongstrethWTJr., ManolioTA, O'LearyDH, BurkeGL, et al. (2007). White matter grade and ventricular volume on brain MRI as markers of longevity in the cardiovascular health study. Neurobiol Aging, 28:1307-1315.16857296 10.1016/j.neurobiolaging.2006.06.010

[20] KerberKA, WhitmanGT, BrownDL, BalohRW (2006). Increased risk of death in community-dwelling older people with white matter hyperintensities on MRI. J Neurol Sci, 250:33-38.16889799 10.1016/j.jns.2006.06.022

[21] LevyRM, SteffensDC, McQuoidDR, ProvenzaleJM, MacFallJR, KrishnanKR (2003). MRI lesion severity and mortality in geriatric depression. Am J Geriatr Psychiatry, 11:678-682.14609809 10.1176/appi.ajgp.11.6.678

[22] van der HolstHM, van UdenIW, TuladharAM, de LaatKF, van NordenAG, NorrisDG, et al. (2016). Factors Associated With 8-Year Mortality in Older Patients With Cerebral Small Vessel Disease: The Radboud University Nijmegen Diffusion Tensor and Magnetic Resonance Cohort (RUN DMC) Study. JAMA Neurol, 73:402-409.26831360 10.1001/jamaneurol.2015.4560

[23] FuS, LeungLY, WangY, RaulliAO, KallmesDF, KinsmanKA, et al. (2019). Natural Language Processing for the Identification of Silent Brain Infarcts From Neuroimaging Reports. JMIR Med Inform, 7:e12109.31066686 10.2196/12109PMC6524454

[24] LeungLY, FuS, LuetmerPH, KallmesDF, MadanN, WeinsteinG, et al. (2021). Agreement between neuroimages and reports for natural language processing-based detection of silent brain infarcts and white matter disease. BMC Neurol, 21:189.33975556 10.1186/s12883-021-02221-9PMC8111708

[25] KentDM, LeungLY, PuttockEJ, WangAY, LuetmerPH, KallmesDF, et al. (2022). Development of Parkinson Disease and Its Relationship with Incidentally Discovered White Matter Disease and Covert Brain Infarction in a Real-World Cohort. Ann Neurol, 92:620-630.35866711 10.1002/ana.26458PMC9489676

[26] Meinel TR, Triulzi CB, Kaesmacher J, Mujanovic A, Pasi M, Leung LY, et al. (2023). Management of covert brain infarction survey: A call to care for and trial this neglected population. European Stroke Journal:23969873231187444. 10.1177/23969873231187444 PMC10683731 37427426

[27] VermeerSE, PrinsND, den HeijerT, HofmanA, KoudstaalPJ, BretelerMM (2003). Silent brain infarcts and the risk of dementia and cognitive decline. N Engl J Med, 348:1215-1222.12660385 10.1056/NEJMoa022066

[28] WardlawJM, SmithEE, BiesselsGJ, CordonnierC, FazekasF, FrayneR, et al. (2013). Neuroimaging standards for research into small vessel disease and its contribution to ageing and neurodegeneration. Lancet Neurol, 12:822-838.23867200 10.1016/S1474-4422(13)70124-8PMC3714437

[29] MunafòMR, TillingK, TaylorAE, EvansDM, Davey SmithG (2018). Collider scope: when selection bias can substantially influence observed associations. International journal of epidemiology, 47.29040562 10.1093/ije/dyx206PMC5837306

[30] WardlawJM, DebetteS, JokinenH, De LeeuwF-E, PantoniL, ChabriatH, et al. (2021). ESO Guideline on covert cerebral small vessel disease. European Stroke Journal, 6:CXI-CLXII.34414301 10.1177/23969873211012132PMC8370079

