# Supplementary material for: Mortality Outcomes in a Large Population with and without Covert Cerebrovascular Disease
Source: Aging Dis. 2024 Feb 11;16(1):512–9. doi: 10.14336/AD.2024.0211 (PMC11745435; doi:10.14336/AD.2024.0211)
Supplement: Supplementary file 1 [file AD-16-1-512-s.pdf]

Mortality Outcomes in a Large Population with and without Covert Cerebrovascular Disease

Úna Clancy, Eric J. Puttock, Wansu Chen, William Whiteley, Ellen M. Vickery, Lester Y. Leung, Patrick H. Luetmer, David F. Kallmes, Sunyang Fu, Chengyi Zheng, Hongfang Liu, David M. Kent

© 2024. Clancy U et al. Published online at http://www.aginganddisease.org/EN/10.14336/AD.2024.0211

Table of Contents

Supplementary Figure 1. Flow chart of participant selection. 3
Supplementary Figure 2. Mortality-free probability in patients (a) with vs without id-CBI and (b) with vs without id-WMH over 144 months of follow-up. 4
Supplementary Table 1. Crude and adjusted hazard ratios (HRs) for mortality by id-CBI and id-WMH. 5
Supplementary Table 2. Crude and adjusted hazard ratios (HR) for mortality by id-CBI and id-WMH for patients with follow up longer than 1 year (n=211,206). 5
Supplementary Table 3. Mortality rate and crude and adjusted hazard ratios (HR) of id-WMH and risk of mortality by severity and modality (n=241,028). 6
Supplementary Table 4. Crude and adjusted effects on mortality for all potential risk factors in the multivariate model. 6
Supplementary Table 5. Mortality rate in patients whose follow-up was longer than 1 year, overall and in subgroups (n=211,206). 8
Supplementary Table 6. Cause-specific mortality rate (n=241,028). 9
Supplementary Table 7. Crude and adjusted effects on cause-specific mortality for all potential risk factors in the multivariate models. 10

© 2024. Clancy U et al. Published online at http://www.aginganddisease.org/EN/10.14336/AD.2024.0211

Supplementary Figure 1. Flow chart of participant selection.

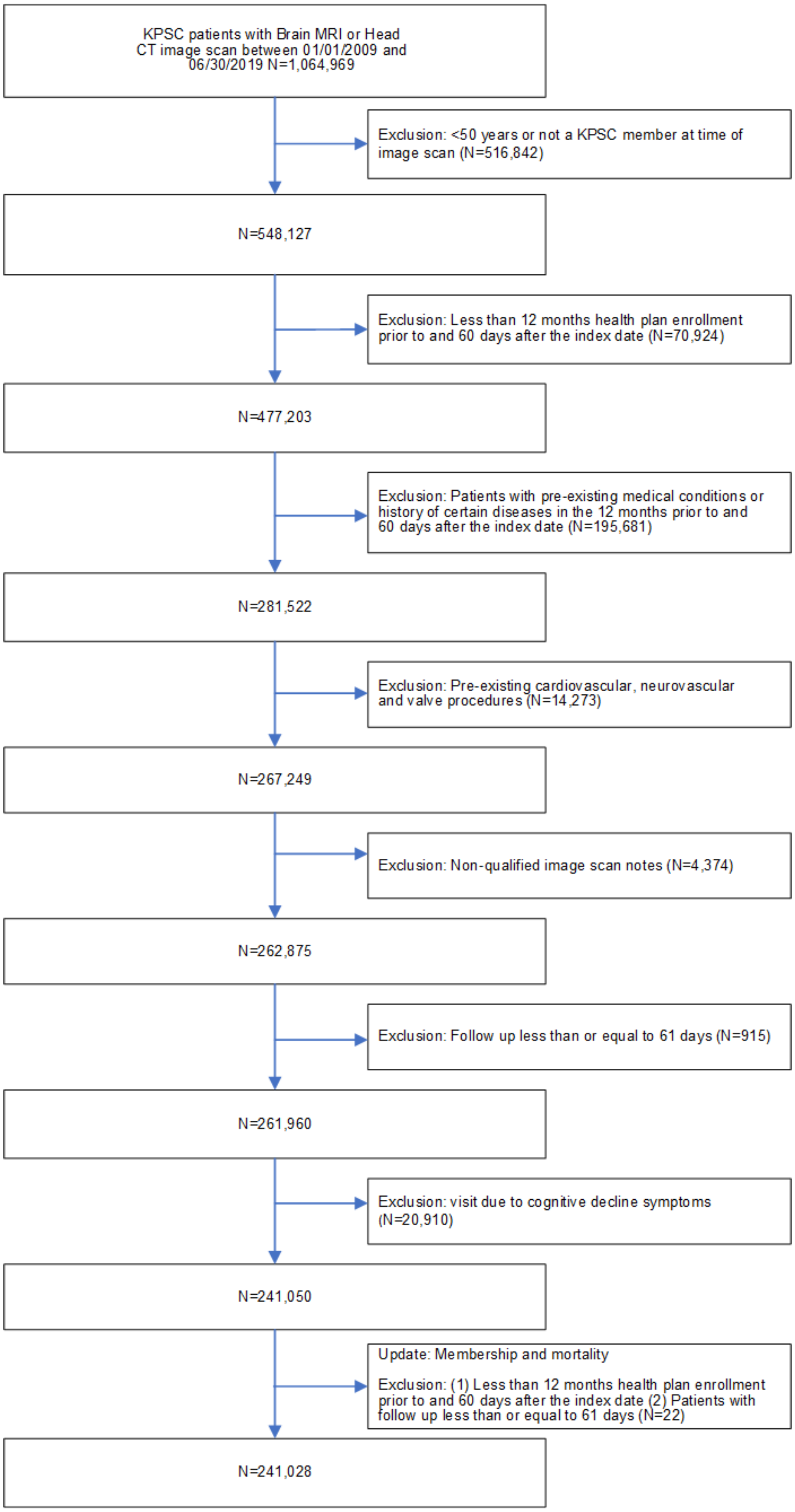

© 2024. Clancy U et al. Published online at http://www.aginganddisease.org/EN/10.14336/AD.2024.0211

Supplementary Figure 2. Kaplan-Meier plots of Mortality-free probability in patients (a) with vs without id-CBI and (b) with vs without id-WMH over 144 months of follow-up. CBI = covert brain infarcts; id = incidentally detected; WMH = white matter hyperintensities

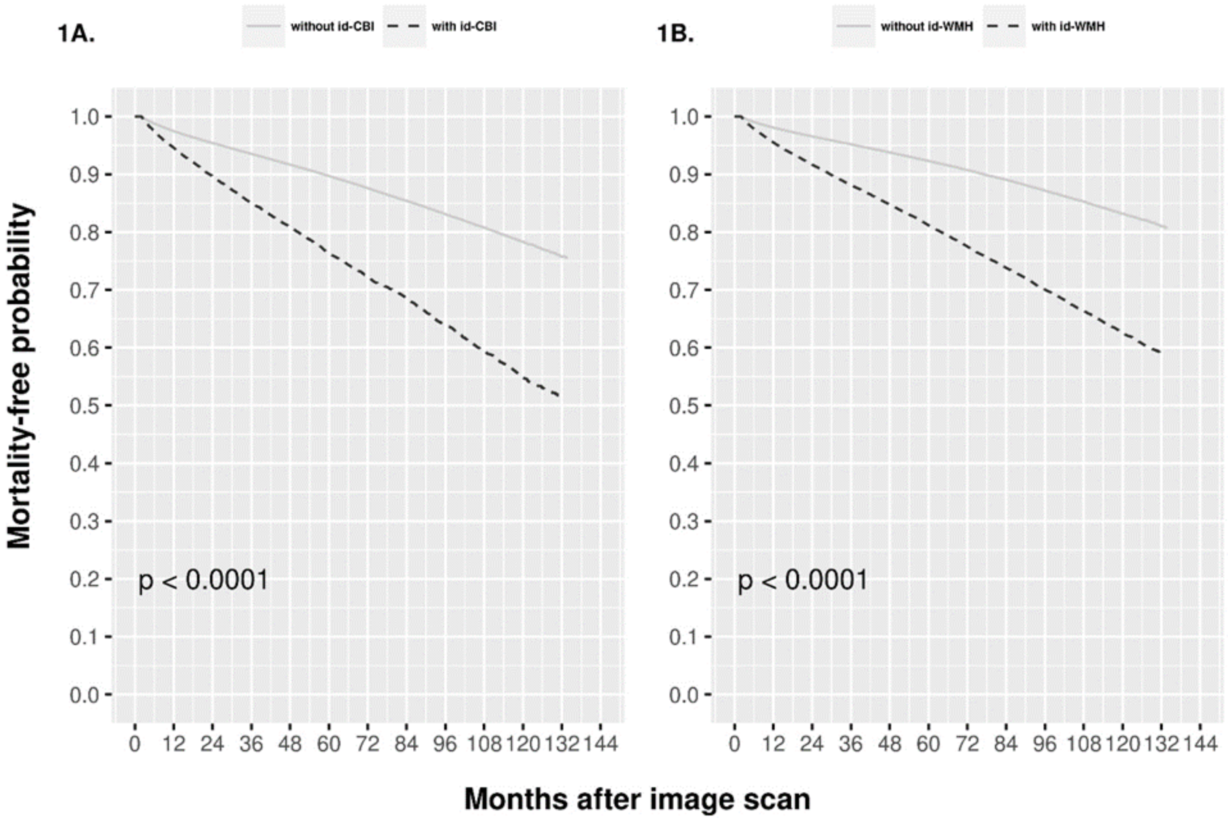

Supplementary Figure 3. Kaplan-Meier plot of mortality-free probability by id-CBI location and modality. CBI = covert brain infarcts; id = incidentally-discovered.

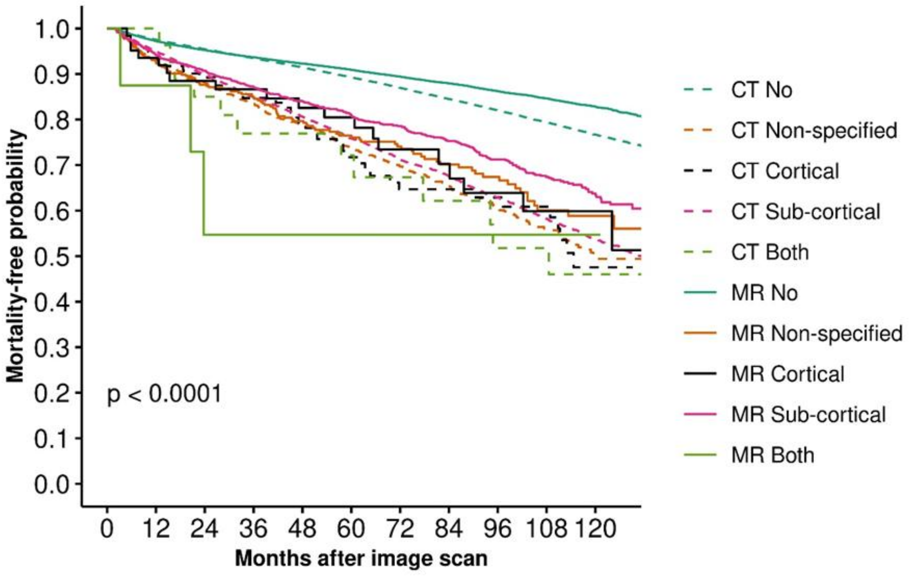

© 2024. Clancy U et al. Published online at http://www.aginganddisease.org/EN/10.14336/AD.2024.0211

SUPPLEMENTARY DATA

Supplementary Table 1. Crude and adjusted hazard ratios (HRs) for mortality by id-CBI and id-WMH.

	HR	95% CI
Crude		
id-CBI	2.42	(2.33, 2.51)
id-WMH	2.56	(2.50, 2.62)
Adjusted (varied by age and modality)		
id-CBI		
Age Mean 64.9	1.27	(1.19, 1.36)
id-WMH		
MRI, Age Mean 64.9	1.20	(1.13, 1.27)
CT, Age Mean 64.9	1.34	(1.28, 1.39)

id-CBI: incidentally-discovered covert brain infarction; id-WMH: incidentally-discovered white matter hyperintensities.
The multivariable model included age, sex, race/ethnicity, atrial fibrillation, carotid atherosclerosis, congestive heart failure, coronary artery disease, diabetes mellitus, hypercholesterolemia, hypertension, peripheral arterial disease, ever tobacco use, mean systolic blood pressure in 1 year, antiplatelet use, stain use, depression, exercise (hour/per week), BMI (kg/m^2), modality, Charlson comorbidity index, age*id-CBI, age*id-WMH, and modality*-idWMH. Refer to Supplementary Table 4 for HRs of all the covariates

Supplementary Table 2. Crude and adjusted hazard ratios (HR) for mortality by id-CBI and id-WMH for patients with follow up longer than 1 year (n=211,206).

	HR	95% CI
Crude		
id-CBI	2.49	(2.39, 2.60)
id-WMH	2.62	(2.56, 2.70)
Adjusted (varied by age and modality)		
id-CBI		
Age Mean 64.9	1.31	(1.21, 1.41)
id-WMH		
MRI, Age Mean 64.9	1.25	(1.17, 1.34)
CT, Age Mean 64.9	1.35	(1.29, 1.42)

id-CBI: incidentally-discovered covert brain infarction; id-WMH: incidentally-discovered white matter hyperintensities.
The multivariable model included age, sex, race/ethnicity, atrial fibrillation, carotid atherosclerosis, congestive heart failure, coronary artery disease, diabetes mellitus, hypercholesterolemia, hypertension, peripheral arterial disease, ever tobacco use, mean systolic blood pressure in 1 year, antiplatelet use, stain use, depression, exercise (hour/per week), BMI (kg/m^2), Charlson comorbidity index, modality, age*id-CBI, age*id-WMH, and modality*id-WMH. Crude and adjusted HRs of all other risk factors included in the model are not shown.

SUPPLEMENTARY DATA

Supplementary Table 3. Mortality rate and crude and adjusted hazard ratios (HR) of id-WMH and risk of mortality by severity and modality (n=241,028).

id-WMH Severity	Number of patients	Average f/u, years	Number of events	Mortality rate/1,000 person years (95% CI)	Crude HR (95% CI)	Adjusted HR[a] (95% CI)
MRI						
None (ref)	29,448	4.51	1,910	14.4 (13.7, 15.0)	1.00	1.00
Mild WMH	20,609	4.38	2,191	24.3 (23.3, 25.3)	1.69 (1.59, 1.80)	1.13 (1.06, 1.20)
Moderate WMH	5,567	4.57	940	36.9 (34.6, 39.4)	2.57 (2.38, 2.78)	1.24 (1.15, 1.34)
Severe WMH	1,768	4.98	399	45.3 (41.0, 49.9)	3.13 (2.81, 3.49)	1.24 (1.11, 1.38)
Not specified	5,082	4.59	685	29.3 (27.2, 31.6)	2.04 (1.87, 2.23)	1.15 (1.05, 1.26)
CT						
None	141,653	4.55	12,222	19.0 (18.6, 19.3)	1.33 (1.26, 1.39)	1.01 (0.97, 1.06)
Mild id-WMH	20,161	4.25	4,517	52.7 (51.2, 54.2)	3.70 (3.51, 3.90)	1.18 (1.12, 1.25)
Moderate id-WMH	3,724	3.94	1,358	92.6 (87.8, 97.6)	6.52 (6.08, 6.99)	1.39 (1.29, 1.49)
Severe id-WMH	1,958	3.93	739	95.9 (89.2, 103.0)	6.74 (6.20, 7.34)	1.45 (1.33, 1.59)
Not specified	11,058	4.06	3,152	70.1 (67.7, 72.6)	4.92 (4.65, 5.20)	1.31 (1.24, 1.39)

id-CBI: incidentally-discovered covert brain infarction; id-WMH: incidentally-discovered white matter hyperintensities.
[a]Adjusted for age group, sex, race/ethnicity, atrial fibrillation, carotid atherosclerosis, congestive heart failure, coronary artery disease, diabetes mellitus, hypercholesterolemia, hypertension, peripheral arterial disease, ever tobacco use, mean systolic blood pressure in 1 year, antiplatelet use, stain use, depression, exercise (hour/per week), BMI (kg/m2), Charlson comorbidity index, and id-CBI.

Supplementary Table 4. Crude and adjusted effects on mortality for all potential risk factors in the multivariate model.

	HR	95% CI
Crude		
Age group (continuous), every 1 year	1.09	(1.09, 1.09)
Sex (reference=Female)	1.43	(1.40, 1.47)
Race/ ethnicity (reference=Non-Hispanic) white)		
Asian and Pacific Islander	0.53	(0.51, 0.56)
African American	0.87	(0.84, 0.90)
Hispanic	0.56	(0.54, 0.57)
Multiple/Other/Unknown	0.41	(0.35, 0.47)
Atrial fibrillation (AF)	3.63	(3.51, 3.74)
Carotid atherosclerosis	2.40	(2.22, 2.61)
Congestive heart failure (CHF)	4.97	(4.82, 5.13)
Coronary artery disease (CAD)	2.54	(2.47, 2.61)
Diabetes mellitus (DM)	1.81	(1.76, 1.85)
Hypercholesterolemia (HC)	1.26	(1.23, 1.30)
Hypertension (HTN)	2.69	(2.61, 2.78)
Peripheral arterial disease (PAD)	3.25	(3.13, 3.36)
Tobacco use (ever)	1.83	(1.79, 1.87)
Systolic blood pressure	1.01	(1.01, 1.02)
Statin	1.36	(1.33, 1.39)
Antiplatelet	1.76	(1.69, 1.84)
Depression	1.06	(1.03, 1.09)
Exercise (hour/per week)	0.87	(0.86, 0.87)
BMI (kg/m^2)	0.96	(0.95, 0.96)
Charlson Comorbidity (ref=0)		
1	1.66	(1.59, 1.73)
2	3.00	(2.89, 3.12)

© 2024. Clancy U et al. Published online at http://www.aginganddisease.org/EN/10.14336/AD.2024.0211

3-4	5.37	(5.18, 5.57)
5+	13.49	(13.04, 13.96)
MRI (reference=CT)	0.79	(0.77, 0.81)
id-CBI	2.42	(2.33, 2.51)
id-WMH	2.56	(2.50, 2.62)
Adjusted		
Sex (reference=Female)	1.39	(1.36, 1.43)
race/ ethnicity (reference=Non-Hispanic) white)		
Asian and Pacific Islander	0.68	(0.65, 0.71)
African American	0.92	(0.89, 0.95)
Hispanic	0.80	(0.78, 0.83)
Multiple/Other/Unknown	0.80	(0.70, 0.92)
Atrial fibrillation (AF)	1.26	(1.22, 1.31)
Carotid atherosclerosis	0.85	(0.78, 0.92)
Congestive heart failure (CHF)	1.37	(1.32, 1.42)
Coronary artery disease (CAD)	0.97	(0.94, 0.999)
Diabetes mellitus (DM)	0.82	(0.79, 0.84)
Hypercholesterolemia (HC)	0.81	(0.78, 0.83)
Hypertension (HTN)	1.16	(1.12, 1.20)
Peripheral arterial disease (PAD)	1.02	(0.99, 1.06)
Tobacco use (ever)	1.27	(1.24, 1.30)
Systolic blood pressure	1.002	(1.001, 1.003)
Statin	0.78	(0.76, 0.80)
Antiplatelet	1.03	(0.99, 1.08)
Depression	1.06	(1.03, 1.10)
Exercise (hour/per week)	0.91	(0.90, 0.92)
BMI (kg/m^2)	0.97	(0.96, 0.97)
Charlson comorbidity index (ref=0)		
1	1.54	(1.48, 1.61)
2	2.27	(2.18, 2.37)
3-4	3.52	(3.38, 3.67)
5+	8.71	(8.36, 9.08)
id-CBI		
Age Mean 64.9	1.27	(1.19, 1.36)
id-WMH		
MRI, Age Mean 64.9	1.20	(1.13, 1.27)
CT, Age Mean 64.9	1.34	(1.28, 1.39)

id-CBI: incidentally-discovered covert brain infarction; id-WMH: incidentally-discovered white matter hyperintensities.
The multivariable model included age, sex, race/ethnicity, atrial fibrillation, carotid atherosclerosis, congestive heart failure, coronary artery disease, diabetes mellitus, hypercholesterolemia, hypertension, peripheral arterial disease, ever tobacco use, mean systolic blood pressure in 1 year, antiplatelet use, stain use, depression, exercise (hour/per week), BMI (kg/m^2), modality, Charlson comorbidity index, age*id-CBI, age*id-WMH, and modality*id-WMH.

© 2024. Clancy U et al. Published online at http://www.aginganddisease.org/EN/10.14336/AD.2024.0211

SUPPLEMENTARY DATA

Supplementary Table 5. Mortality rate in patients whose follow-up was longer than 1 year, overall and in subgroups (n=211,206).

Patient characteristics	Patients with id-CBI (n=9,904)			Patient with id-WMH (n=60,746)			Patients with both id-CBI and Id-WMH (n=5,424)			Patients without id-CCD[a] (n= 145,980)		
	Avg f/u, yrs	No. events	Mortality rate (95% CI)	Avg f/u, yrs	No. events	Mortality rate (95% CI)	Avg f/u, yrs	No. events	Mortality rate (95% CI)	Avg f/u, yrs	No. events	Mortality rate (95% CI)
All	4.33	2,451	57.1 (54.9,59.4)	4.05	10,888	44.3 (43.4,45.1)	4.05	1,629	74.2 (70.7,77.9)	4.25	10,054	16.2 (15.9,16.5)
Age group (yrs)												
50-59	4.56	90	13.5 (10.9,16.5)	4.18	486	10.2 (9.3,11.1)	4.47	42	21.6 (15.8,29.0)	4.18	1,839	6.4 (6.1,6.7)
60-69	4.53	340	28.5 (25.6,31.6)	4.18	1,580	21.6 (20.6,22.7)	4.21	183	36.7 (31.7,42.3)	4.30	2,674	13.2 (12.7,13.7)
70-79	4.59	823	54.9 (51.2,58.7)	4.16	3,609	45.6 (44.1,47.1)	4.33	518	61.2 (56.1,66.6)	4.41	3,126	31.1 (30.0,32.2)
80+	3.66	1,198	128.7 (121.6,136.1)	3.59	5,213	113.2 (110.1,116.3)	3.55	886	135.2 (126.5,144.3)	4.02	2,415	84.5 (81.2,88.0)
Sex												
Female	4.53	1,321	50.8 (48.1,53.6)	4.15	6,263	40.3 (39.3,41.3)	4.26	886	66.8 (62.5,71.3)	4.34	5,433	13.8 (13.5,14.2)
Male	4.06	1,130	66.9 (63.1,70.9)	3.88	4,625	51.2 (49.7,52.6)	3.76	743	85.5 (79.5,91.8)	4.10	4,621	20.3 (19.7,20.9)
Race/ethnicity												
Non-Hisp. White	4.28	1,340	66.5 (63.0,70.1)	4.08	6,447	51.0 (49.8,52.3)	4.01	943	85.0 (79.7,90.5)	4.32	5,187	20.8 (20.2,21.3)
Asian/PI	4.58	195	38.6 (33.4,44.3)	4.10	907	31.0 (29.0,33.1)	4.11	119	48.4 (40.3,57.7)	4.40	864	11.1 (10.4,11.8)
African Am.	4.53	394	59.7 (54.0,65.8)	4.20	1,399	49.2 (46.7,51.8)	4.25	255	76.5 (67.6,86.3)	4.53	1,335	17.7 (16.8,18.7)
Hispanic	4.25	510	47.5 (43.5,51.7)	3.93	2,070	34.9 (33.4,36.5)	4.04	307	62.6 (55.9,69.9)	4.06	2,605	12.5 (12.0,13.0)
Other[b]	3.32	12	33.5 (18.3,56.8)	3.45	65	24.5 (19.0,31.0)	2.83	5	31.0 (10.1,72.3)	3.49	63	7.6 (5.9,9.6)
Imaging Modality												
CT	4.31	2,074	60.6 (58.0,63.3)	3.88	7,831	63.1 (61.7,64.5)	3.95	1,316	85.5 (81.0,90.2)	4.25	8,823	17.2 (16.8,17.6)
MRI	4.41	377	43.4 (39.2,48.0)	4.23	3,057	25.1 (24.2,26.0)	4.29	313	47.7 (42.7,53.2)	4.25	1,231	11.5 (10.8,12.1)

PI: Pacific islander; id-CBI: incidentally-discovered covert brain infarction; id-WMH: incidentally-discovered white matter hyperintensities; id-CCD: incidentally-discovered covert cerebrovascular disease.
[a]id-CBI or id-WMH; [b]Multiple, other or unknown race/ethnicity

© 2024. Clancy U et al. Published online at http://www.aginganddisease.org/EN/10.14336/AD.2024.0211

Supplementary Table 6. Cause-specific mortality rate (n=241,028).

Cause	Patients with id-CBI (n=11,328)		Patient with id-WMH (n=69,927)		Patients with both id-CBI and id-WMH (n=6,284)		Patients without id-CCD[a] (n=166,057)	
	Number of events	Mortality rate (95% CI)	Number of events	Mortality rate (95% CI)	Number of events	Mortality rate (95% CI)	Number of events	Mortality rate (95% CI)
Avg. length of y/u, yrs	4.57		4.30		4.27		4.53	
Underlying cause of death								
CVD	977	18.9 (17.7,20.1)	3,960	13.2 (12.8,13.6)	667	24.8 (23.0,26.8)	2,851	3.8 (3.7,3.9)
HD	629	12.1 (11.2,13.1)	2,673	8.9 (8.6,9.2)	430	16.0 (14.6,17.6)	2,047	2.7 (2.6,2.8)
CeVD	211	4.1 (3.6,4.7)	776	2.6 (2.4,2.8)	148	5.5 (4.7,6.5)	449	0.6 (0.5,0.7)
PD	50	1.0 (0.7,1.3)	272	0.9 (0.8,1.0)	32	1.2 (0.8,1.7)	201	0.3 (0.2,0.3)
Dementia	309	6.0 (5.3,6.7)	1,308	4.3 (4.1,4.6)	226	8.4 (7.4,9.6)	608	0.8 (0.7,0.9)
Cancer	859	16.6 (15.5,17.7)	4,743	15.8 (15.3,16.2)	551	20.5 (18.9,22.3)	5,579	7.4 (7.2,7.6)
Other	830	16.0 (15.0,17.1)	3,548	11.8 (11.4,12.2)	534	19.9 (18.3,21.6)	3,708	4.9 (4.8,5.1)
Contributing cause of death								
CVD	2,058	39.7 (38.0,41.5)	8,774	29.2 (28.6,29.8)	1,381	51.4 (48.8,54.2)	7,322	9.7 (9.5,10.0)
HD	1,661	32.1 (30.6,33.6)	7,064	23.5 (22.9,24.0)	1,113	41.4 (39.1,43.9)	5,878	7.8 (7.6,8.0)
CeVD	366	7.1 (6.4,7.8)	1,243	4.1 (3.9,4.4)	258	9.6 (8.5,10.8)	758	1.0 (0.9,1.1)
PD	71	1.4 (1.1,1.7)	404	1.3 (1.2,1.5)	46	1.7 (1.3,2.3)	324	0.4 (0.4,0.5)
Dementia	504	9.7 (8.9,10.6)	2,069	6.9 (6.6,7.2)	372	13.9 (12.5,15.3)	992	1.3 (1.2,1.4)
Cancer	962	18.6 (17.4,19.8)	5,177	17.2 (16.7,17.7)	618	23.0 (21.3,24.9)	5,957	7.9 (7.7,8.1)
Other	1,986	38.3 (36.7,40.1)	8,929	29.7 (29.1,30.3)	1,332	49.6 (47.0,52.3)	8,571	11.4 (11.2,11.6)

CVD: Cardiovascular disease (I00-I99); HD: Heart disease (I00-I09, I11, I13, I20-I51); CeVD: Cerebrovascular disease (I60-I69); PD: Parkinson's disease (G20,G21); Dementia (F01, F03, G30); Cancer (C00-C97); Other (all other deaths with known causes); 1.1% of deaths had unknown causes; id-CBI: incidentally-discovered covert brain infarction; id-WMH: incidentally-discovered white matter hyperintensities; id-CCD: incidentally-discovered covert cerebrovascular disease.
[a]id-CBI or id-WMH.

© 2024. Clancy U et al. Published online at http://www.aginganddisease.org/EN/10.14336/AD.2024.0211

Supplementary Table 7. Crude and adjusted effects on cause-specific mortality for all potential risk factors in the multivariate models.

	HR and 95% CI		
	CVD	CeVD	Dementia
Crude			
Age group (continuous), every 1 year	1.13 (1.13, 1.13)	1.14 (1.13, 1.15)	1.20 (1.19, 1.20)
Sex (reference=Female)	1.40 (1.34,1.47)	0.92 (0.82,1.04)	0.94 (0.86,1.03)
Race/ethnicity (reference=Non-Hispanic white)			
Asian and Pacific Islander	0.45 (0.41,0.50)	0.64 (0.53,0.77)	0.40 (0.34,0.48)
African American	0.89 (0.83,0.96)	0.97 (0.82,1.14)	0.76 (0.67,0.87)
Hispanic	0.47 (0.44,0.50)	0.59 (0.51,0.67)	0.44 (0.39,0.49)
Multiple/Other/Unknown	0.39 (0.30,0.53)	0.45 (0.24,0.88)	0.30 (0.16,0.55)
Atrial fibrillation (AF)	6.77 (6.42,7.13)	5.44 (4.77,6.20)	3.61 (3.19,4.07)
Carotid atherosclerosis	3.67 (3.22,4.19)	3.05 (2.17,4.29)	2.76 (2.06,3.68)
Congestive heart failure (CHF)	9.75 (9.25,10.27)	4.34 (3.71,5.07)	3.26 (2.84,3.76)
Coronary artery disease (CAD)	4.16 (3.96,4.37)	2.73 (2.40,3.10)	2.25 (2.02,2.50)
Diabetes mellitus (DM)	1.99 (1.90,2.09)	1.73 (1.55,1.94)	1.35 (1.22,1.48)
Hypercholesterolemia (HC)	1.54 (1.46,1.63)	1.53 (1.35,1.74)	1.18 (1.07,1.30)
Hypertension (HTN)	5.65 (5.22,6.10)	4.65 (3.93,5.51)	3.22 (2.86,3.62)
Peripheral arterial disease (PAD)	4.76 (4.48,5.07)	4.11 (3.52,4.79)	3.00 (2.61,3.45)
Tobacco use (ever)	1.69 (1.61,1.77)	1.27 (1.14,1.42)	1.12 (1.03,1.23)
Systolic blood pressure	1.03 (1.03,1.03)	1.04 (1.03,1.04)	1.03 (1.03,1.03)
Statin	1.60 (1.53,1.68)	1.44 (1.30,1.61)	1.25 (1.14,1.36)
Antiplatelet	2.49 (2.31,2.69)	2.13 (1.76,2.59)	1.65 (1.39,1.97)
Depression	1.05 (0.99,1.12)	1.03 (0.90,1.19)	1.06 (0.95,1.19)
Exercise (hour/per week)	0.85 (0.83,0.86)	0.89 (0.86,0.92)	0.85 (0.83,0.86)
BMI (kg/m^2)	0.96 (0.95,0.96)	0.92 (0.91,0.94)	0.96 (0.95,0.96)
Charlson comorbidity index (reference=0)			
1	1.70 (1.57,1.83)	1.57 (1.33,1.85)	1.25 (1.10,1.42)
2	2.80 (2.60,3.03)	2.33 (1.96,2.76)	2.20 (1.94,2.49)
3-4	5.60 (5.23,5.99)	4.34 (3.72,5.06)	2.99 (2.63,3.38)
5+	8.76 (8.17,9.40)	5.18 (4.36,6.17)	2.88 (2.46,3.38)
MRI (reference=CT)	0.39 (0.36,0.41)	0.44 (0.37,0.51)	0.40 (0.35,0.46)
id-CBI	3.13 (2.93,3.35)	3.84 (3.32,4.46)	3.56 (3.15,4.02)
id-WMH	3.27 (3.12,3.43)	3.98 (3.56,4.45)	5.04 (4.59,5.52)
Adjusted			
Age group (continuous), every 1 year	NA	1.10 (1.09, 1.11)	NA
Sex (reference=Female)	1.53 (1.46, 1.61)		1.29 (1.17, 1.42)
Race/ethnicity (reference=Non-Hispanic white)			
Asian and Pacific Islander	0.66 (0.60, 0.73)		0.54 (0.46, 0.65)
African American	0.95 (0.88, 1.02)		1.01 (0.88, 1.15)
Hispanic	0.73 (0.68, 0.77)		0.76 (0.67, 0.85)
Multiple/Other/Unknown	0.98 (0.74, 1.30)		0.71 (0.38, 1.32)
Atrial fibrillation (AF)	1.72 (1.62, 1.83)	1.98 (1.72, 2.29)	1.20 (1.06, 1.36)
Carotid atherosclerosis	1.15 (1.01, 1.32)		
Congestive heart failure (CHF)	2.46 (2.31, 2.62)	1.20 (1.01, 1.43)	
Coronary artery disease (CAD)	1.23 (1.16, 1.30)		
Diabetes mellitus (DM)	1.12 (1.05, 1.18)		1.32 (1.19, 1.47)
Hypercholesterolemia (HC)	0.85 (0.80, 0.91)		0.85 (0.77, 0.94)
Hypertension (HTN)	1.67 (1.54, 1.83)	1.43 (1.19, 1.73)	
Peripheral arterial disease (PAD)	1.30 (1.22, 1.39)	1.40 (1.19, 1.64)	
Tobacco use (ever)	1.16 (1.10, 1.22)		
Systolic blood pressure	1.01 (1.01, 1.01)	1.02 (1.01, 1.02)	
Statin	0.84 (0.79, 0.89)	0.85 (0.76, 0.96)	
Antiplatelet	1.19 (1.10, 1.29)		
Depression	1.13 (1.06, 1.20)	1.17 (1.02, 1.35)	1.38 (1.23, 1.54)
Exercise (hour/per week)	0.92 (0.90, 0.93)	0.95 (0.92, 0.98)	0.89 (0.87, 0.92)

© 2024. Clancy U et al. Published online at http://www.aginganddisease.org/EN/10.14336/AD.2024.0211

BMI (kg/m^2)	0.98 (0.97, 0.98)	0.96 (0.95, 0.98)	0.93 (0.92, 0.94)
Charlson comorbidity index (reference=0)			
1	1.14 (1.05, 1.23)	1.14 (0.96, 1.35)	
2	1.27 (1.17, 1.37)	1.19 (0.997, 1.42)	
3-4	1.65 (1.53, 1.79)	1.62 (1.37, 1.92)	
5+	1.96 (1.79, 2.15)	1.88 (1.55, 2.29)	
MRI (reference=CT)	0.58 (0.54, 0.63)	0.62 (0.52, 0.73)	0.61 (0.53, 0.70)
id-CBI		1.55 (1.33, 1.80)	
Age Mean 64.9	1.62 (1.42, 1.86)		1.75 (1.27, 2.41)
id-WMH	1.34 (1.27, 1.41)	1.62 (1.44, 1.84)	
Age Mean 64.9			3.13 (2.53, 3.87)

CVD: Cardiovascular disease (I00-I99); CeVD: Cerebrovascular disease (I60-I69); Dementia (F01, F03, G30); id-CBI: incidentally-discovered covert brain infarction; id-WMH: incidentally-discovered white matter hyperintensities

© 2024. Clancy U et al. Published online at http://www.aginganddisease.org/EN/10.14336/AD.2024.0211
